# Behavioral Dentistry in the Digital Age: A Review of Biofeedback Occlusal Splint Therapy

**DOI:** 10.7759/cureus.106620

**Published:** 2026-04-07

**Authors:** Akshita Chipper, Swapnali Mhatre, Reema Srichand, Saniya Kulkarni, Uttam Shetty, Mridula Joshi

**Affiliations:** 1 Prosthodontics and Crown and Bridge and Implantology, Bharati Vidyapeeth (Deemed to be University) Dental College and Hospital, Navi Mumbai, IND

**Keywords:** behavior-modifying approach, biofeedback occlusal splint, bruxism, occlusal splint therapy, temporomandibular disorders

## Abstract

Occlusal splints have long been employed as conservative therapeutic devices in the management of temporomandibular disorders (TMDs), bruxism, and occlusal dysfunctions. Traditionally fabricated from hard acrylic, these splints function primarily through passive mechanical protection and require consistent patient compliance for effectiveness.

This review outlines the evolution of occlusal splint therapy, with an emphasis on recent advancements in digital fabrication and biofeedback integration, and evaluates their clinical relevance in improving treatment outcomes. It begins by summarizing conventional classifications, clinical indications, and the biomechanical principles underlying splint therapy. Recent innovations are discussed, including the emergence of biofeedback-enabled splints incorporating embedded electromyographic or pressure sensors to identify parafunctional activity and deliver real-time stimuli for neuromuscular modulation. Simultaneously, progress in computer-aided design and manufacturing (CAD-CAM), 3D printing, and smart material technologies has enhanced splint precision, durability, and patient-specific customization. Additionally, mobile health platforms and remote monitoring tools play a growing role in promoting patient adherence and facilitating post-delivery follow-up.

Occlusal splint therapy is undergoing a technological transformation, shifting from passive mechanical devices to interactive, data-driven therapeutic tools. These modern systems integrate wearable sensor technology, digital fabrication, and behavioral feedback to enable more personalized and effective management of TMDs and sleep bruxism. While early results are promising, robust clinical trials and long-term studies are needed to validate their efficacy and support their integration into routine clinical practice.

## Introduction and background

Occlusal splints, commonly referred to as bite guards or occlusal appliances, are removable devices used in dentistry for both therapeutic and diagnostic purposes. Traditionally employed to manage bruxism and protect dental structures from occlusal trauma, their use has expanded to include the treatment of temporomandibular disorders (TMDs), myofascial pain dysfunction, and as supportive therapy in full-mouth rehabilitation cases. These appliances aim to redistribute occlusal forces, promote muscle relaxation, and stabilize the temporomandibular joint (TMJ) by altering the patient’s occlusal relationship temporarily or permanently, depending on the indication [1,2].

TMDs are a prevalent group of musculoskeletal conditions that affect the jaw joint and associated structures. Symptoms may include joint pain, clicking sounds, limited mandibular movement, and muscle tenderness, often affecting a patient’s quality of life. Occlusal splints are considered a conservative and reversible intervention, making them a valuable first-line approach in managing such conditions. Despite their common use, the selection and design of splints require individualized clinical judgment based on accurate diagnosis and symptomatology [2].

In recent years, significant technological advancements have redefined the therapeutic potential of occlusal splints. Among the most notable innovations are biofeedback occlusal splints, which incorporate sensor technology to monitor and respond to nocturnal bruxism or clenching activity in real time. These devices use electromyographic (EMG) or pressure sensors to detect parafunctional activity and subsequently deliver a mild vibratory or auditory stimulus to alert the patient, promoting subconscious muscle relaxation and helping reduce the frequency and intensity of episodes over time [3,4]. Unlike passive appliances, biofeedback splints offer an interactive, behavior-modifying approach, merging dentistry with wearable technology and neuromodulation principles [5].

Parallel to this, the integration of digital workflows, including computer-aided design and manufacturing (CAD/CAM) design, intraoral scanning, and 3D printing, has improved the precision, reproducibility, and comfort of splint therapy [2]. Materials have also evolved, with flexible, thermoadaptive polymers and biocompatible printable resins offering enhanced durability and patient acceptance. Moreover, emerging tools such as AI-based occlusal analysis systems and mobile-app-connected splints are enabling clinicians to remotely monitor patient compliance and parafunctional patterns [4].

As these innovations shift occlusal splints from passive mechanical protectors to smart therapeutic devices, it becomes increasingly important for clinicians to understand their design principles, mechanisms of action, and clinical indications. This review explores the occlusal splints while placing particular emphasis on the advancements in biofeedback technology and digital manufacturing, highlighting their potential to improve outcomes in the management of bruxism and TMDs.

Types of occlusal splints

Conventional Occlusal Splints: Foundation of Therapy

Based on material composition: Hard occlusal splints are made from heat-cured acrylic resins or thermoplastics and provide rigid support. They are indicated for severe bruxism or cases involving significant occlusal wear. An example includes Michigan-type splints. Hard splints have demonstrated greater efficacy in reducing muscle activity during nocturnal bruxism compared to soft splints. Soft Occlusal Splints: Fabricated from resilient materials like silicone or ethylene-vinyl acetate, these splints offer cushioning and comfort. They are typically used in mild bruxism or pediatric cases. However, studies have shown they may paradoxically increase muscle activity in some individuals due to rebound effects [6]. 

Based on coverage: Full-arch coverage splints encompass all teeth in one arch (usually maxillary) and distribute occlusal forces evenly. Commonly used for long-term management, examples include stabilization splints and flat-plane splints. Anterior Coverage Splints: These splints cover only the anterior teeth (usually canines and incisors) and function by limiting posterior occlusal contact to reduce muscle hyperactivity. Devices like the NTI-tss and anterior bite plates fall into this category. However, long-term use may result in posterior open bite due to disclusion [7]. 

Based on occlusal design and function: Stabilization splints (flat plane splints) provide even, simultaneous contact in centric relation, aiming to reduce muscle activity and protect the dentition. They are often indicated for myofascial pain and TMD and are among the most frequently prescribed occlusal appliances. Repositioning splints alter the mandibular position to a therapeutic location and are used in cases of internal derangement or joint disc displacement. However, they require careful monitoring due to the risk of occlusal changes with prolonged use [8]. Guidance splints (canine or anterior guidance), designed to guide mandibular movements, minimize occlusal interferences during lateral and protrusive excursions and are particularly useful in functional occlusal disorders.

Based on duration of use: Interim (temporary) splints fabricated for short-term use (e.g., in acute TMD episodes or post-trauma) are often prefabricated or made chairside and may lack the precision or durability of long-term appliances. Definitive (long-term) splints, custom-made from durable materials and tailored to the patient’s occlusal anatomy, are indicated for chronic bruxism or long-standing TMD. They are adjusted precisely for long-term therapeutic goals. Their mechanisms include neuromuscular relaxation, joint unloading, proprioceptive modulation, and occlusal harmony. Despite their wide usage, traditional splints often suffer from limitations in adjustability, reproducibility, and long-term patient compliance [9]. 

Digitally and CAD/CAM-Fabricated Occlusal Splints

The integration of digital technologies such as intraoral scanning, CAD and CAM, has significantly advanced the fabrication of occlusal splints, offering improvements in precision, efficiency, and reproducibility. Intraoral scanners enable the acquisition of highly accurate digital impressions, thereby eliminating common distortions associated with conventional elastomeric materials. This enhances the marginal fit and occlusal accuracy of the final appliance [10]. CAD software platforms such as Exocad and 3Shape facilitate detailed occlusal analysis, virtual articulation, and customization of splint design based on the patient's anatomical and functional parameters. These programs allow for dynamic occlusal mapping, which aids in identifying interferences and optimizing occlusal contacts prior to manufacturing. Splints can be produced via additive (3D printing) or subtractive (milling) manufacturing techniques. Additive manufacturing, using biocompatible resins like NextDent Ortho Rigid or Formlabs Dental LT Clear, allows for rapid, cost-effective fabrication of durable, esthetic, and patient-specific appliances. These materials comply with ISO standards and offer excellent mechanical strength, optical clarity, and long-term wear resistance. Their application in both laboratory and chairside workflows significantly reduces turnaround time while maintaining clinical precision [11].

Occlusal analysis systems such as T-Scan provide dynamic, real-time data on occlusal force distribution and timing. When integrated into the digital workflow, this diagnostic data enables biomechanically optimized splint designs that enhance force redistribution and improve therapeutic outcomes and patient comfort. Moreover, digital workflows facilitate easy reproduction; in the event of loss or damage, splints can be re-fabricated from archived STL files without the need for a new clinical impression. Reduced chairside time is another notable advantage, as the high initial accuracy minimizes the need for occlusal adjustments during delivery. Collectively, these advancements reflect a paradigm shift in occlusal splint therapy, from analog craftsmanship to digitally driven precision, enhancing both clinician efficiency and patient outcomes.

Biofeedback Occlusal Splints

Biofeedback occlusal splints represent a significant advancement in the therapeutic management of sleep bruxism (SB) and related parafunctional activities by incorporating interactive, neuromodulatory mechanisms. These devices are designed to provide real-time feedback through embedded sensors, typically EMG or pressure-based, that detect clenching or grinding episodes during sleep. Upon detecting abnormal muscle activity, the splint delivers a mild vibratory or auditory stimulus, prompting subconscious inhibition of the behavior and promoting neuromuscular relaxation [12] (Table 1).

**Table 1:** Difference between conventional and biofeedback occlusal splints

Feature	Traditional splint	Biofeedback splint
Feedback mechanism	None	Real-time(audio/tactile)
Muscle relaxation	Passive	Active
Data recording	No	Yes
Customization	Manual	Sensor-driven
Compliance monitoring	No	Yes

Emerging materials and smart splint integration

Recent advancements in material science and digital technologies have transformed the clinical potential of occlusal splints, enhancing both functionality and patient experience. Novel material compositions and smart integration approaches now enable splints to serve not just as mechanical protectors, but as interactive, personalized therapeutic devices. Thermoadaptive polymers, with their capacity for temperature-sensitive deformation, offer enhanced intraoral adaptation and patient comfort. These materials adjust subtly to thermal variations in the oral environment, improving marginal integrity and minimizing discomfort during prolonged wear. Dual-laminate or bimaterial designs, combining a rigid external layer for occlusal stability with a resilient inner surface for cushioning, have demonstrated superior wear resistance and improved tolerability. Such configurations are particularly beneficial in patients with high bite forces or sensitivity to rigid appliances [13]. 

Moreover, mobile app integration and cloud-based platforms now allow for remote patient monitoring, usage compliance tracking, and in some devices, direct control or feedback delivery. These features are especially valuable in the management of chronic conditions such as bruxism and TMDs, offering clinicians’ actionable data while engaging patients in their own care. Together, these advancements are redefining the therapeutic utility of occlusal splints, positioning them at the intersection of dentistry, material science, and digital health.

## Review

Materials and methods

Search Strategy

A structured literature search was conducted following Preferred Reporting Items for Systematic Reviews and Meta-Analyses (PRISMA) guidelines across PubMed, Scopus, and Web of Science for studies published between January 2010 and December 2024.

The search combined MeSH terms and keywords: (“occlusal splints” OR “dental splints”) AND (“biofeedback” OR “electromyography” OR “EMG”) AND (“sleep bruxism” OR “bruxism” OR “temporomandibular disorders” OR “TMD”).

Only English-language, human studies, and peer-reviewed articles were included. Reference lists of selected articles were also screened manually.

Eligibility Criteria

Studies were included if they involved adult patients with SB or TMDs and evaluated biofeedback-based occlusal splints. Randomized controlled trials, clinical studies, pilot studies, and systematic reviews were considered.

Exclusion criteria included animal studies, case reports, conference abstracts, and studies lacking relevant outcome measures.

Study Selection

After removal of duplicates, titles and abstracts were screened independently by two reviewers. Full texts of eligible studies were assessed, and disagreements were resolved by consensus.

PRISMA Flowchart

**Figure 1:**
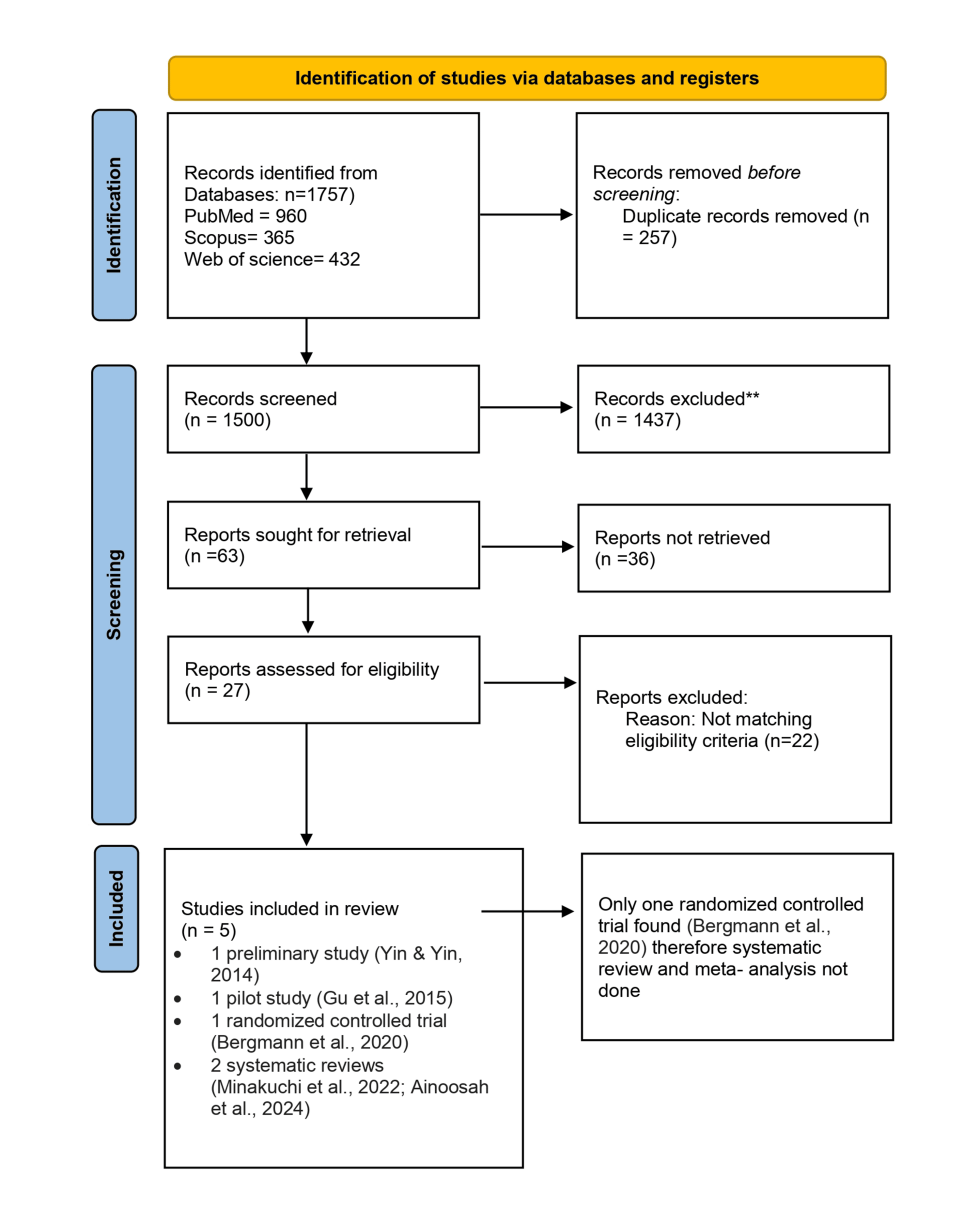
PRISMA chart PRISMA: Preferred Reporting Items for Systematic Reviews and Meta-Analyses

The search yielded 1,757 records, of which 27 were screened for full-text eligibility. Five studies [14-18] met the inclusion criteria and were included in the final analysis for qualitative review (Figure 1).

Studies included in qualitative synthesis (n = 5) were as follows: one preliminary study (Yin & Yin, 2014) [14], one pilot study (Gu et al., 2015) [15], one randomized controlled trial (Bergmann et al., 2020) [16], and two systematic reviews (Minakuchi et al., 2022; Ainoosah et al., 2024) [17,18]. As there were not enough randomized controlled trials (only one was found), quantitative review was not possible.

Biofeedback therapy

Biofeedback therapy (BFT) has emerged as a valuable non-invasive modality in the multidisciplinary management of TMDs. TMDs often involve hyperactivity of the masticatory muscles, leading to pain, dysfunction, and reduced quality of life. Biofeedback targets these maladaptive neuromuscular patterns by enabling patients to become aware of and consciously regulate muscle activity. In the context of TMDs, EMG biofeedback is most commonly employed. This technique records muscle tension in real-time and presents it to the patient through visual or auditory signals. By recognizing patterns of excessive clenching or grinding, individuals are trained to voluntarily reduce muscle tension, mitigating strain on the TMJ and surrounding musculature [19].

Several clinical studies have demonstrated that incorporating biofeedback into TMD management protocols can lead to significant reductions in pain intensity, frequency of parafunctional habits, and overall muscle tension [15,20]. Unlike pharmacologic treatments, biofeedback focuses on behavioral retraining and does not carry the risk of systemic side effects, making it particularly suitable for long-term use or for patients who prefer non-drug therapies [20].

Additionally, when integrated with occlusal splints or cognitive-behavioral therapy, biofeedback can enhance treatment outcomes by addressing both the physical and psychological components of TMDs. Portable biofeedback devices, and more recently, biofeedback-enabled occlusal splints, have expanded the accessibility of this approach, allowing for therapy to continue beyond the clinical setting.

Mechanism of action

Biofeedback occlusal splints operate through a closed-loop system that facilitates neuromuscular re-education by continuously monitoring muscle activity and delivering real-time sensory stimuli to disrupt parafunctional habits. The mechanism of action involves the following components.

Muscle Activity Detection

The primary mechanism of biofeedback occlusal splints involves that the splint is embedded with EMG sensors or pressure-sensitive components that detect involuntary clenching or grinding episodes. A predefined threshold is programmed into the system to differentiate between normal and excessive muscle activity. This ensures that the device is responsive to clinically relevant levels of parafunction while minimizing false-positive signals [15].

Immediate Biofeedback Response

Upon detection of abnormal muscle activity, the device delivers a real-time feedback stimulus aimed at interrupting the ongoing bruxism episode. This stimulus can be delivered in several formats: Vibration alerts: A mild buzzing sensation prompts the patient to relax the involved musculature. Auditory signals: Beeps or tones act as an external cue to raise awareness during sleep. Mild electrical stimulation: Low-intensity impulses are used to gently disrupt ongoing contractions and promote neuromuscular relaxation [21]. This immediate response creates an associative link between the muscle activity and the external cue, initiating a feedback loop that encourages behavioral regulation.

Behavioral Modification Over Time

With repeated night use, patients begin to develop subconscious awareness of their bruxing behavior. This promotes behavioral adaptation, wherein patients progressively learn to suppress or reduce clenching and grinding episodes, even in the absence of the device. Over time, this can lead to a sustained reduction in bruxism frequency and intensity, reflecting neuromuscular retraining rather than mere mechanical prevention.

Data Monitoring and Adjustments

**Figure 2:**
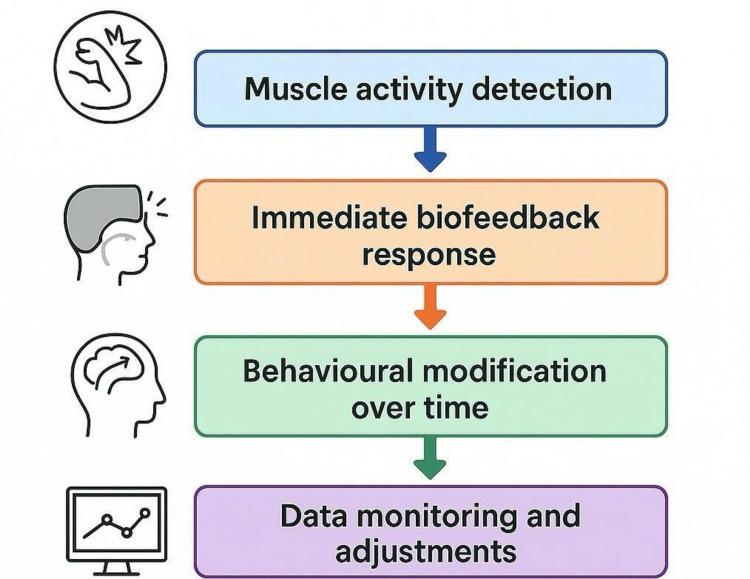
Mechanism of a biofeedback occlusal splint Image Credit: Dr Akshita Chipper. Created using Canva (Canva Pty Ltd., Sydney, Australia).

Advanced biofeedback splint systems often allow for cloud-based data storage and monitoring of bruxism activity. Clinicians can adjust parameters such as detection sensitivity and feedback intensity based on the patient’s therapeutic response and tolerance. This personalization enhances treatment efficacy and improves long-term adherence (Figure 2).

Types of Biofeedback Occlusal Devices

**Figure 3:**
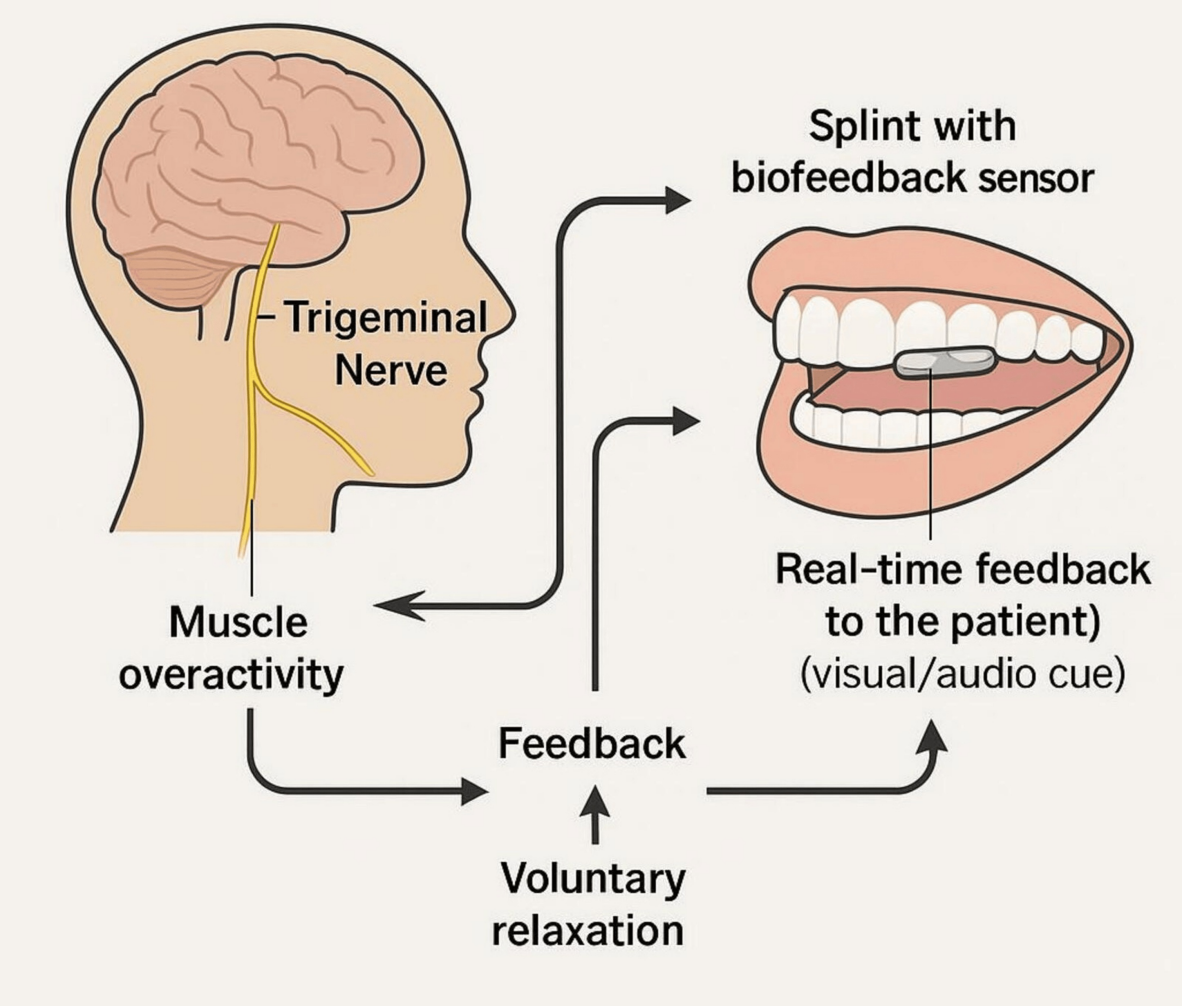
Diagrammatic representation of occlusal devices used for biofeedback Image Credit: Dr Akshita Chipper. Created using Canva (Canva Pty Ltd., Sydney, Australia).

Biofeedback-based devices for bruxism deliver real-time stimuli to interrupt excessive masticatory muscle activity. These systems vary by design, sensor integration, and feedback mechanism. GrindCare™: A head-worn device with EMG sensors delivering low-level electrical stimulation during bruxism episodes. It reduces event frequency without disturbing sleep significantly [22]. BruxOff™: This combines EMG monitoring with mobile app feedback (auditory/vibratory), offering real-time alerts via Bluetooth for awake and SB [23]. SleepGuard™: This uses an auditory feedback loop triggered by clenching activity to condition muscle relaxation during sleep. SMART Splint™: Intraoral splints are embedded with EMG or pressure sensors providing feedback directly. Integration with mobile apps enables remote monitoring and adjustment. These systems represent a shift toward personalized, minimally invasive behavioral therapy for bruxism (Figure 3).

Biofeedback splints offer a progressive and interactive approach to managing bruxism and TMDs, shifting away from the traditional passive role of occlusal appliances. Unlike conventional splints that merely act as protective barriers, biofeedback devices engage the patient in behavioral modification by providing real-time sensory cues, such as gentle vibrations or sound alerts, when abnormal muscle activity is detected. This facilitates neuromuscular retraining and promotes long-term habit correction. Being non-invasive and drug-free, these splints present a safe alternative to pharmacologic interventions, particularly for patients seeking holistic or minimally invasive therapies. The use of feedback mechanisms enhances patient self-awareness, encouraging greater control over oral habits and fostering better compliance.

Clinical evidence supports their effectiveness in reducing the frequency, duration, and intensity of bruxism episodes, which can subsequently alleviate associated symptoms like jaw pain, headaches, and muscle fatigue [16]. Additionally, the integration of these devices with mobile health applications enables remote monitoring, compliance tracking, and individualized adjustments, further supporting personalized care. By minimizing nocturnal parafunctional activity, biofeedback splints contribute to improved sleep quality and daily functioning. Over time, consistent use may lead to sustained behavioral change, reducing the need for long-term appliance use or adjunctive interventions.

Limitations of biofeedback splints

While biofeedback splints offer a progressive approach to managing SB and TMDs, several limitations must be acknowledged. A major barrier is their relatively high cost compared to traditional occlusal appliances, primarily due to the integration of sensor technologies and digital connectivity. This financial burden can restrict accessibility, particularly in underserved populations or among patients without insurance coverage. Another challenge lies in patient compliance, as the effectiveness of BFT depends heavily on consistent use. Sensory cues, such as vibrations or auditory alerts, although essential for therapeutic action, may be perceived as disruptive during sleep, especially during the initial adaptation phase. Moreover, the technological sensitivity of these devices introduces potential issues with calibration, sensor alignment, or software reliability, which may compromise the accuracy of feedback signals. There is also a lack of universally accepted clinical guidelines delineating which patient subgroups would benefit most from this modality.

While biofeedback splints show promise for managing primary bruxism, their role in more complex or multifactorial cases involving TMDs, anxiety-related clenching, or chronic orofacial pain remains under investigation. Maintenance requirements and device longevity further complicate their use; embedded electronics demand regular servicing, and the potential for reduced durability compared to traditional acrylic splints may necessitate more frequent replacement. Additionally, these devices may not be suitable for all individuals, particularly those with neurological disorders, hypersensitivity to stimuli, or limited comfort with technology. As such, careful patient selection and individualized treatment planning are critical to optimizing outcomes [24]. Biofeedback splints may not be suitable for pediatric patients, individuals with cognitive impairments, or those with severe sleep disorders. Furthermore, patients with pacemakers or sensitivity to electrical stimulation should be carefully evaluated before using EMG-based systems. 

SB is a movement disorder associated with sleep, marked by repetitive jaw muscle activity such as teeth clenching or grinding. While conventional management strategies such as occlusal splints are widely used, recent advances in wearable technologies have introduced BFT as a promising behavioral intervention. Research shows that biofeedback splints can reduce bruxism episodes and EMG amplitude, improve sleep quality and reduce morning orofacial pain, decrease masseter muscle hyperactivity in short-term trials, enhance patient awareness and engagement in therapy [15,16]. This literature review of available data conducted to critically evaluates the current evidence supporting the efficacy of biofeedback-based approaches, particularly biofeedback splints, in comparison to traditional occlusal devices. 

Initial clinical investigations have explored the utility of BFT as a non-invasive modality for the management of SB, particularly when integrated into wearable devices or occlusal splint systems. In a pilot study by Gu et al. (2015) [15], the effectiveness of a miniaturized wireless biofeedback device was compared to that of conventional occlusal splints. Twenty-four participants diagnosed with SB were randomly assigned to two groups: one receiving BFT (GTB) and the other treated with standard occlusal splints (GTO). Bruxism activity was assessed using a standardized analysis program (TRMY1.0), which recorded the frequency and duration of bruxism episodes during eight-hour sleep sessions. After six and 12 weeks of intervention, the GTB group demonstrated a statistically significant reduction in both outcome measures, while no such improvements were observed in the GTO group. The biofeedback device was well tolerated, with minimal sleep disruption, indicating good compliance and acceptability.

Supporting these findings, a preliminary study by Yin and Yin (2014) [14] evaluated a closed-loop biofeedback system incorporating a maxillary splint with an embedded strain gauge sensor and a wearable vibratory alert unit. The system continuously monitored occlusal force during sleep and delivered real-time vibratory feedback in response to excessive or prolonged parafunctional activity. Over a 24-week treatment period, participants experienced a reduction of over 60% in the frequency and more than 70% in the duration of bruxism episodes-both statistically significant improvements, sustained throughout the intervention period.

These studies collectively highlight the potential of wearable biofeedback systems in facilitating neuromuscular adaptation and behavioral modification through subconscious sensory feedback mechanisms [14,15]. By promoting muscle relaxation and reducing parafunctional load during sleep, such devices may offer a more dynamic and behaviorally guided alternative to traditional occlusal splints. However, limitations such as small sample sizes, short-to-moderate follow-up durations, and a focus on patients with mild bruxism underscore the need for larger, rigorously controlled trials to confirm these preliminary outcomes and establish broader clinical applicability.

Further evidence for the efficacy of biofeedback-based interventions is provided by a randomized controlled clinical trial conducted by Bergmann et al. (2020) [16], which evaluated the impact of a full-occlusion biofeedback (BFB) splint compared to a conventional adjusted occlusal splint (AOS) in patients with SB and TMD-associated pain over a 12-week period. The BFB splint incorporated a feedback mechanism that responded to excessive occlusal force without disturbing sleep architecture, enabling subconscious behavioral modulation. Patients in the BFB group demonstrated statistically significant reductions in both the frequency and duration of bruxism episodes, along with substantial improvements in facial muscle pain and self-reported well-being. Importantly, while the reduction in bruxism duration persisted even after discontinuation of the biofeedback intervention, the frequency of episodes returned to baseline, indicating partial retention of therapeutic benefit. In contrast, the AOS group exhibited comparatively limited improvements in both pain symptoms and bruxism metrics. These findings highlight the superior clinical performance of biofeedback splints in attenuating nocturnal parafunctional activity and mitigating TMD-related discomfort. The reduction in masticatory muscle overload likely contributes to both symptom relief and the prevention of long-term temporomandibular joint degeneration. Nonetheless, the study emphasizes the need for further research involving larger cohorts and patients with higher baseline pain intensities to optimize treatment protocols and enhance generalizability.

Systematic review and comparative evidence

The clinical relevance of BFT in the management of SB has been further reinforced by a systematic review conducted by Minakuchi et al. (2022) [17]. The review focused on BFT integrated within occlusal splints, which function by detecting rhythmic masticatory muscle activity (RMMA) and delivering real-time sensory cues-typically vibratory or auditory-to disrupt involuntary muscular contractions during sleep. Among these modalities, vibration-based biofeedback splints consistently demonstrated favorable outcomes, including significant reductions in EMG activity and bruxism episode frequency across multiple randomized controlled trials.

These splints are notable for their non-invasive nature and lack of pharmacologic side effects, making them a viable and well-tolerated option for a wide patient population. However, while short-term results are encouraging, the review identified mixed findings regarding the sustainability of these effects. In several studies, bruxism activity gradually returned to baseline levels following discontinuation of therapy, suggesting the need for sustained or intermittent use to maintain therapeutic benefits. This highlights the importance of personalized treatment plans and ongoing monitoring in optimizing long-term outcomes.

Complementing this, a comparative analysis by Ainoosah et al. (2024) [18] examined the relative efficacy of various occlusal splint designs. Full-coverage biofeedback splints with integrated vibratory mechanisms demonstrated superior performance in reducing both the frequency and duration of bruxism episodes. These splints were also associated with enhanced muscle relaxation, significant decreases in patient-reported facial pain, and improved overall quality of life, benefits likely driven by their ability to provide immediate, subconscious neuromuscular feedback during sleep.

Conventional rigid stabilization splints were effective in mitigating TMJ discomfort and providing occlusal protection but were generally less effective than biofeedback designs in reducing EMG activity. Soft splints, while often preferred for comfort, showed variable outcomes; in some cases, they were linked to increased clenching behavior. Additionally, digitally fabricated splints-especially those made from advanced materials like polyetheretherketone-offered improvements in fit, durability, and production efficiency, though comparative data on their clinical efficacy remain limited [18].

Both reviews emphasized a key limitation in the current body of evidence: substantial heterogeneity in study designs, splint configurations, diagnostic tools, and outcome assessment methods [17,18]. This variability hinders the ability to draw definitive conclusions and limits generalizability across patient populations. Accordingly, there is a strong need for large-scale, standardized clinical trials employing objective diagnostic modalities-such as polysomnography or BiteStrip®-based EMG monitoring-to further validate and refine the clinical application of biofeedback-enhanced splints in the management of SB.

The current body of evidence suggests that BFT, particularly when integrated into occlusal splint designs, offers a promising and non-invasive approach for managing sleep bruxism [14,16-18]. Compared to conventional splint therapy, biofeedback splints demonstrate superior performance in reducing bruxism activity and associated myofascial pain. However, long-term efficacy data remain limited, and heterogeneity in study design hinders broad clinical applicability. Future research should focus on standardized methodologies, long-term follow-up, and patient stratification to optimize treatment protocols and guide individualized therapy.

## Conclusions

Biofeedback occlusal splints mark a transformative advancement in the management of bruxism and TMDs, offering a shift from conventional passive devices to interactive, behavior-modifying therapeutic tools. By integrating real-time sensor technology, these splints actively engage patients in neuromuscular retraining, facilitating a more dynamic and personalized treatment approach. The incorporation of mobile health platforms and remote monitoring further aligns with modern trends in digital healthcare, enhancing compliance and continuity of care. Current evidence supports their effectiveness in reducing parafunctional muscle activity, improving patient-reported outcomes, and promoting long-term habit modification. However, challenges such as cost, user adaptability, and technical reliability remain barriers to widespread clinical implementation. Future large-scale, well-controlled clinical studies and standardized assessment protocols will be crucial to establishing their long-term efficacy and defining their optimal role within interdisciplinary management strategies.
